# *Anopheles gambiae* Actively Metabolizes Uric Acid Following *Plasmodium* Infection to Limit Malaria Parasite Survival

**DOI:** 10.3389/fphys.2021.821869

**Published:** 2022-01-24

**Authors:** Hyeogsun Kwon, Ryan Smith

**Affiliations:** Department of Entomology, Iowa State University, Ames, IA, United States

**Keywords:** mosquito physiology, host-pathogen interactions, uric acid, urate oxidase, malaria, *Anopheles gambiae*, *Plasmodium berghei*

## Abstract

Characterizing the physiological changes that accompany malaria parasite infection of the mosquito host is crucial to our understanding of vectorial capacity in *Anopheles* mosquitoes, yet has not fully been explored. In this study, we examine the role of uric acid metabolism in the mosquito, *Anopheles gambiae*, following malaria parasite infection. We demonstrate that levels of uric acid are significantly decreased in the excreta and the mosquito at 24 and 48 h post-*Plasmodium* infection when compared to controls fed on naïve mouse blood. When we examine the expression of well-known enzymes responsible for uric acid metabolism, we see a significant increase in both *urate oxidase* (*UO*) and *allatoicase* (*ALLC*) expression following *Plasmodium* infection. Targeting the essential first step in uric acid metabolism by silencing *UO* resulted in elevated levels of uric acid, enhancing malaria parasite survival. With implications from other insect systems that bacteria can modulate UO expression, we examined the possibility that the mosquito microbiota and its expansion following blood-feeding may contribute to increased UO levels. However, there was no difference in uric acid metabolism between septic and aseptic mosquitoes, indicating that the mosquito microbiome is not associated with the manipulation of *UO* expression. Together, our study provides new evidence that *Plasmodium* infection causes the mosquito host to actively metabolize uric acid by increasing *UO* expression to limit *Plasmodium* oocyst survival, suggesting that nitrogen metabolism is an essential pathway in defining mosquito vector competence.

## Introduction

Mosquito blood-feeding provides essential resources for egg production and enables mosquitoes to serve as efficient vectors to acquire and transmit several mosquito-borne pathogens. Most notably, *Plasmodium* parasites cause the devastating public health and socioeconomic impacts of malaria throughout the world (World Health Organization, 2019).

Independent of the presence of a pathogen, blood-feeding also represents a major physiological event. Digestion of the protein-rich blood meal and its conversion into nutritional resources represents a major metabolic event for the mosquito, stimulating several physiological processes, including vitellogenesis (Attardo et al., 2005), immune induction (Upton et al., 2015; Reynolds et al., 2020b), and epithelial turnover (Taracena et al., 2018). In addition, enzymes that promote blood digestion result in the release of heme, which can lead to an increase in levels of reactive oxygen species (ROS) and potential cytotoxic effects (Whiten et al., 2018). To overcome this oxidative stress, mosquitoes maintain physiological homeostasis by detoxifying the accumulated reactive molecules *via* peroxidases and catalase activities (Molina-Cruz et al., 2008; Champion and Xu, 2017). In addition, excessive amino acids are catabolized into uric acid (Von Dungern and Briegel, 2001; Scaraffia et al., 2008), providing additional antioxidants to maintain physiological homeostasis (Molina-Cruz et al., 2008), or are converted by the enzymes urate oxidase (UO), allantoinase (ALLN), allantoicase (ALLC) into urea through the uricolytic pathway as nitrogenous waste (Scaraffia et al., 2008; Isoe and Scaraffia, 2013).

Several studies provide evidence that this regulation of oxidative homeostasis is integral to mosquito vector competence, where increased levels of ROS and subsequent nitration in the mosquito host have been shown to limit malaria parasite survival (Luckhart et al., 1998; Kumar et al., 2003; Peterson et al., 2007; Molina-Cruz et al., 2008; Eleftherianos et al., 2021). Reciprocally, when increased levels of antioxidants are provided (as either uric acid/urate or ascorbic acid) to *Plasmodium* infected mosquitoes, parasite survival is increased (Peterson et al., 2007; Molina-Cruz et al., 2008). Similarly, in other insect systems, uric acid promotes the success of *Trypanosoma brucei brucei* survival in tsetse flies (MacLeod et al., 2007), and it has been suggested that *Drosophila* actively reduce uric acid levels to limit bacterial growth (Chambers et al., 2012; Lang et al., 2019). Therefore, the physiological and immunological mechanisms of uric acid synthesis and metabolism are essential to our understanding of mosquito vector competence.

Herein, we describe differences in the regulation of uric acid metabolism between blood-fed (naïve) and *Plasmodium*-infected *Anopheles gambiae*, demonstrating that malaria parasite infection promotes increased levels of urate oxidase (UO) activity in the mosquito host. Driving the metabolism of uric acid into urea, we demonstrate that silencing *UO* results in increased uric acid levels, enhancing parasite survival. Taken together, these observations suggest that the mosquito host manipulates uric acid metabolism in response to infection to limit pathogen survival.

## Materials and Methods

### Mosquito Rearing and *Plasmodium* Infection

*Anopheles gambiae* (Keele strain) were reared at 27°C and 80% humidity, with a 14/10-h day/night cycle. Larvae were fed on fish flakes (Tetramin, Tetra), and adult mosquitoes were maintained on 10% sucrose solution.

Female Swiss Webster mice were infected with a mCherry strain of *Plasmodium berghei* as previously described (Kwon et al., 2017; Kwon and Smith, 2019). Parasite infection was quantified at 8 days post-infection by counting oocyst numbers from individual midgut samples using a fluorescent microscope (Nikon Eclipse 50i, Nikon).

### Uric Acid Quantification

*Anopheles gambiae* mosquitoes were challenged by blood-feeding with either a naïve or *P. berghei*-infected mouse. Within 30 min after challenge, fully engorged mosquitoes were collected and placed in a glass vial (20 × 70 mm) containing a small cotton ball applied with 200 μl of 10% sucrose for the first 24 h, and transferred to a new glass vial and kept for additional 24 h. Mosquitoes were maintained at 19°C with 80% relative humidity and a 14/10-h light/dark cycle. Uric acid levels were measured in excreta from individual mosquitoes by the addition of 0.5 ml of 1% lithium carbonate in distilled water to each glass vial to dissolve the solid excreta. To each tube, 2.5 ml of uric acid reagent consisting of a 10:1 mixture of Solution I (377 mM sodium carbonate, 213 mM glycine, 3.1 mM cupric sulfate in distilled water) and Solution II (19 mM neocuproine hydrochloride monohydrate in distilled water) were, respectively added to the resuspended lithium carbonate. The mixture was incubated for 5 min at room temperature to maximize (∼95%) color development as previously described (Handel, 1975; Van Handel and Klowden, 1996). A standard curve was generated using serial dilutions of uric acid in 1% lithium carbonate solution (ranges from 50 μg to 0.1 ug/ml) and was used to determine uric acid concentrations in samples when measured at 450 nm using VERSAmax™ Tunable Microplate Reader (Molecular Devices). Individual mosquito samples at 48 h post-challenge were homogenized in 0.5 ml of 1% lithium carbonate solution to extract uric acid, heated at 80°C for 3 min, and centrifuged at 12,000 × *g* for 10 min (Scaraffia et al., 2008). The supernatant (0.5 ml) was mixed with the uric acid reagent mixture (2.5 ml) to measure uric acid levels as described above.

### Gene Expression Analysis by qRT-PCR

To determine whether differences in uric acid production between blood-fed and *P. berghei* infected mosquitoes display changes in uricolytic pathway gene expression, mosquitoes challenged with either a naïve or *P. berghei*-infected blood meal were collected at 3, 24, and 48 h post-challenge. Mosquitoes (*n* = 15 per treatment at each time point) were used for total RNA isolation using TRIzol (Thermo Fisher Scientific). Total RNA (2 μg) was treated with DNase I (New England Biolabs) and used for cDNA synthesis using the RevertAid First Strand cDNA Synthesis kit (Thermo Fisher Scientific). qRT-PCR analysis was performed using cDNA (1:5 dilution), 500 nM of gene-specific primers and PowerUp™SYBR^®^Green Master Mix (Thermo Fisher Scientific) with the following cycling conditions: 95°C for 10 min, 40 cycles with 95°C for 15 s and 65°C for 60 s. Ribosomal protein S7 transcript levels were used as an internal reference as described previously (Kwon et al., 2017; Kwon and Smith, 2019). A comparative C_*T*_ (2^–ΔΔCt^) method was employed to determine relative transcript abundance for each transcript (Livak and Schmittgen, 2001). Primers used for qRT-PCR are listed in Supplementary Table 1.

### Gene-Silencing *via* RNAi

Since mosquitoes challenged with *P. berghei* exhibited increases of urate oxidase expression and lower amounts of uric acid production as compared to naive blood-fed mosquitoes, the potential role of UO in *Plasmodium* survival was investigated through gene silencing. RNAi experiments were performed as previously described (Kwon et al., 2017; Kwon and Smith, 2019). T7 DNA template was amplified using cDNA prepared from whole mosquitoes collected 24 h post-*P. berghei* infection and T7 primers designed using the E-RNAi web application.^1^ Primers are listed in Supplementary Table 1. Amplified PCR products were gel purified using the Gel DNA Recovery kit (Zymo Research) and dsRNA was prepared using the MEGAscript RNAi kit (Life Technologies) according to the manufacturer’s instructions. After ethanol precipitation, dsRNA was resuspended in nuclease free water to a final concentration of 3 μg/μl. Three to four-day old mosquitoes were cold anesthetized and injected intrathoracically with 69 nl (∼200 ng) of dsRNA per mosquito using a Nanoject III injection system (Drummond Scientific). Silencing efficiency was evaluated 2 days post-injection of dsRNA in whole mosquito samples (15 mosquitoes per treatment). Total RNA isolation, cDNA synthesis and qRT-PCR analysis were performed as described above. To evaluate the effect of gene-silencing on uric acid metabolism and *Plasmodium* survival, mosquitoes at 2 days post-injection of dsRNA were challenged with *P. berghei*. Engorged individual mosquitoes were transferred to a glass vial and kept for 24 h, then transferred to a new glass vial for an additional 24 h to measure uric acid in excreta, as well as in the whole mosquito body at 48 h-post-infection. Samples for the uric acid measurement were prepared as described above. Oocyst survival was determined by counting oocyst numbers at 8 days post-infection.

### Contribution of Midgut Bacteria to Uric Acid Metabolism

To determine whether the expression of *UO* is altered by bacterial abundance in mosquitoes, mosquitoes (*n* = 10 per treatment at each time point) were challenged with either a naive or an infected blood meal and collected at 3 h, 24 h and 48 h post-challenge. Mosquitoes were surface-sterilized in 75% ethanol for 5 min, then washed twice with sterile 1xPBS before used for total RNA isolation and cDNA synthesis as described above. Universal bacterial 16s rRNA primers (Supplementary Table 1) were used for the quantification of bacteria as previously described (Blumberg et al., 2013; Smith et al., 2015; Reynolds et al., 2020a). Newly emerged female mosquitoes (0 to 1-day-old) were maintained on 10% sucrose or a 10% sterile sucrose solution containing antibiotics (100 units/mL of penicillin and 100 μg/mL of streptomycin) for 3 days. The reduction of bacterial 16s rRNA in aseptic mosquitoes (*n* = 10) was determined by qRT-PCR at 3 days post-treatment using the universal bacterial primers. The mosquitoes were challenged with *P. berghei* infection to quantify uric acid in excreta and whole body from individual mosquitoes at 24 h and 48 h post-infection as described above.

## Results

### Uric Acid Levels Differ Between Blood-Fed and *Plasmodium*-Infected Mosquitoes

**FIGURE 1:**
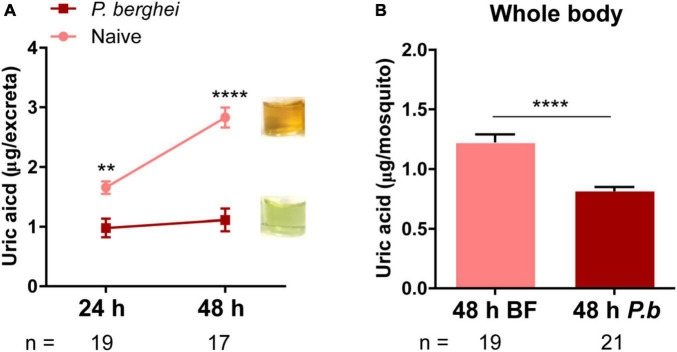
Uric acid levels are reduced in excreta and in whole mosquitoes following *Plasmodium* infection. Uric acid in the excreta of individual mosquitoes was measured at both 24 and 48 h after blood-feeding (naïve) or *P. berghei* infection. *Plasmodium* infection significantly reduced uric acid concentration in excreta when compared to mosquitoes that fed on a naïve blood meal. Inset images display representative images of differences in the intensity of uric acid observed in excreta samples at 48 h-post challenge **(A)**. Data from excreta samples were analyzed using a two-way repeated-measures ANOVA followed by Sidak’s multiple comparisons. Uric acid levels were similarly measured in whole mosquito samples 48 h after blood-feeding (naïve) or *P.* berghei infection **(B)**, where *Plasmodium*-infected mosquitoes retained less uric acid than those provided with a naïve blood meal. Data were analyzed by an unpaired *t*-test. Bar graphs represent mean ± SEM of three independent experiments. Asterisks denote significance (**P < 0.01, ****P < 0.0001). n, number of individual mosquitoes examined. Data analysis was performed using GraphPad Prism 7.

With previous results supporting the role of uric acid as a key antioxidant that supports malaria parasite survival (Peterson et al., 2007; Molina-Cruz et al., 2008), we wanted to examine if uric acid production varied between naïve and infected blood meals. To approach this question, we challenged mosquitoes with a naïve or *P. berghei*-infected mouse and examined levels of uric acid in the excreta of individual mosquitoes at 24 h and 48 h post-challenge. Compared to mosquitoes fed on a naïve blood meal, infected mosquitoes excreted significantly less uric acid at both 24 h and 48 h post-infection (Figure 1A). However, since this only examined excreta, there is the potential that uric acid was instead being retained in the mosquito host. To address this, we examined levels of uric acid in whole mosquitoes, demonstrating that uric acid levels were also similarly reduced in *P. berghei-*infected mosquitoes at 48 h post-challenge (Figure 1B). Together, these observations suggest that uric acid metabolism in the mosquito is altered as a result of *Plasmodium* infection.

### Key Enzymes in Uric Acid Metabolism Are Influenced by *Plasmodium* Infection

**FIGURE 2:**
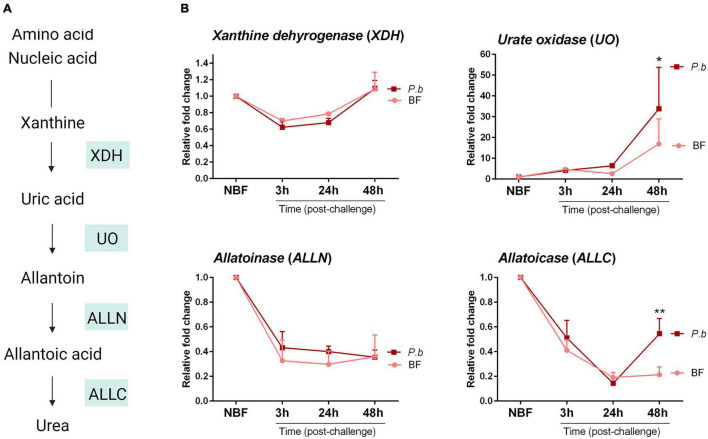
Gene expression analysis of uricolytic pathway enzymes following blood-feeding and *P. berghei* infection. **(A)** Overview of the intermediates and enzymes involved in nitrogen metabolism of amino acids and nucleic acids into urea. Genes encoding key enzymes: *xanthine dehydrogenase* (*XDH*), *urate oxidase* (*UO*), *allatoinase* (*ALLN*), and *allatoicase* (*ALLC*), are shown in shaded boxes. **(B)** Gene expression analysis of *XDH*, *UO, ALLN*, and *ALLC* under sugar-fed (NBF) conditions or 3, 24, and 48 h after feeding on a naïve or *P. berghei*-infected blood meal. Data from three independent experiments are displayed as the mean ± SEM and were analyzed using a two-way repeated-measures ANOVA followed by Sidak’s multiple comparison to determine significant differences. Asterisks denote significance (*P < 0.05, **P < 0.01). Data analysis was performed using GraphPad Prism 7.

Previous studies have closely examined the metabolism of nitrogenous compounds in the uricolytic pathway following mosquito blood-feeding (Scaraffia et al., 2008; Isoe and Scaraffia, 2013), defining key enzymes involved in the production of uric acid and its eventual breakdown into urea (Figure 2A). Given the differences in uric acid levels between blood-fed and infected mosquitoes in Figure 1, we examined how infection may regulate the expression of key enzymes in the uricolytic pathway (Figure 2A). Relative expression levels of *xanthine dehydrogenase* (*XDH*), *urate oxidase* (*UO*), *allatoinase* (*ALLN*) and *allatoicase* (*ALLC*) were analyzed in mosquitoes challenged with a naïve or *Plasmodium*-infected blood meal at 3, 24, and 48 h post-challenge, with time points representing pre-ookinete invasion, ookinete invasion, and post-ookinete invasion, respectively. *UO* and *ALLC* expression was significantly increased in *Plasmodium*-infected mosquitoes 48 h post-infection when compared to mosquitoes fed on naïve blood (Figure 2B), suggesting that ookinete invasion and/or the formation of early oocysts are responsible for these physiological changes. In contrast, there was no difference in the relative expression of *XDH* and *ALLN* (Figure 2B). Since UO is required for the initial step in uric acid metabolism (Figure 2A), its increased expression may account for the reduced uric acid levels following *Plasmodium* infection (Figure 1).

### *Urate oxidase*-Silencing Increases Levels of Uric Acid and Enhances *Plasmodium* Survival

**FIGURE 3:**
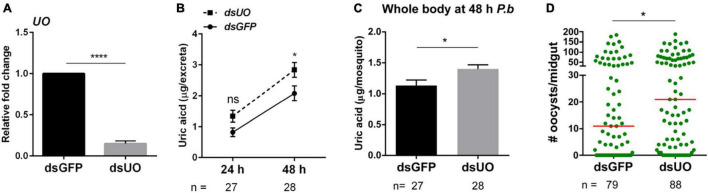
*Urate oxidase* (*UO*) silencing increases uric acid levels and *Plasmodium* oocyst survival. To determine whether accumulation of uric acid in the mosquito promotes the success of oocyst survival, Urate oxidase (*UO*) silencing was validated by qRT-PCR 2 days post-injection of dsRNA **(A)**, resulting in the increase in uric acid measured in excreta **(B)** and whole mosquitoes **(C)** as compared to dsGFP controls. When *UO*-silenced mosquitoes were challenged with *P. berghei*, we observed a significant increase in oocyst survival when compared to GFP controls at 8 days post-infection **(D)**. qRT-PCR and whole mosquito uric acid data were analyzed using an unpaired *t*-test, while uric acid excreta data were analyzed using a two-way repeated-measures ANOVA followed by Sidak’s multiple comparison test. Data are displayed as the mean ± SEM of three independent experiments. Differences in oocyst survival were analyzed using Mann–Whitney analysis, with median oocyst numbers represented by the horizontal red line. Asterisks denote significance (*P < 0.05, ****P < 0.0001). n, number of individual mosquitoes examined. Data analysis was performed using GraphPad Prism 7.

To more closely examine the role of UO on uric acid metabolism and its effects on *Plasmodium* infection, we performed RNAi to silence *UO* expression. Following the injection of dsRNA, *UO* expression was significantly reduced 2 days post-injection (Figure 3A). To confirm the role of UO in uric acid metabolism, we demonstrate that *UO*-silencing results in increased uric acid levels in excreta at 48 h post-infection (Figure 3B) as well as the accumulation of uric acid in *UO*-silenced mosquitoes (Figure 3C). When *UO*-silenced mosquitoes were challenged with *P. berghei* infection, there were significantly higher oocyst numbers (Figure 3D), suggesting that the higher accumulation of uric acid in *UO*-silenced mosquitoes promoted increased oocyst survival similar to previous experiments when uric acid was administered through sugar feeding (Molina-Cruz et al., 2008). Therefore, these findings indicate that mosquito host manipulation of the oxidative environment following infection *via* UO regulation is a novel defense mechanism to decrease *Plasmodium* survival.

### The Mosquito Microbiota Does Not Influence Uric Acid Metabolism

**FIGURE 4:**
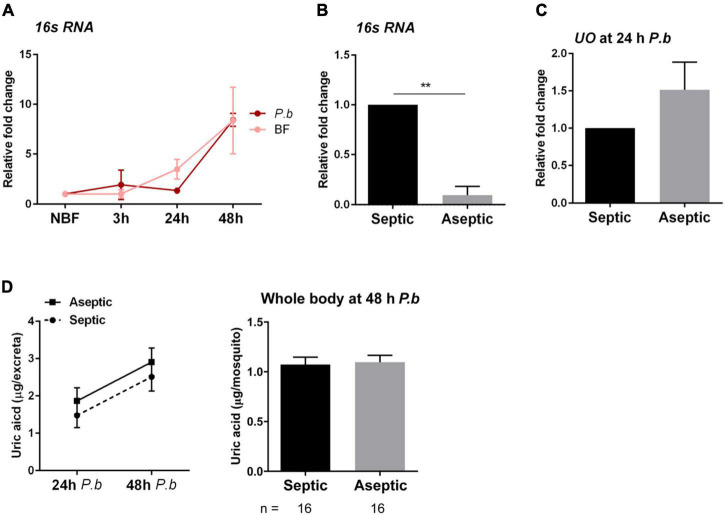
The mosquito microbiota does not influence uric acid production. The impacts of blood-feeding and *P. berghei* infection on the mosquito microbiome were analyzed in whole mosquitoes by the relative quantification of bacterial 16s rRNA *via* qRT-PCR **(A)**. Mosquitoes were examined from sugar-fed (NBF) conditions or 3, 24, and 48 h after feeding on a naïve or *P. berghei*-infected blood meal. To determine the contribution of the mosquito microbiota to the metabolism of uric acid, mosquitoes were treated with antibiotics in 10% sucrose for 3 days (aseptic), and compared to mosquitoes maintained on 10% sucrose alone (septic) through the relative quantification of bacterial 16s rRNA **(B)**. The reduction of microbiota levels did not influence urate oxidase (*UO*) expression **(C)**, or the production of uric acid **(D)**, measured in both the excreta and the whole mosquito. For all experiments, data were from three independent experiments and displayed as the mean ± SEM. Data examining the 16s rRNA levels **(A)** and uric acid in excreta **(D)** were analyzed using a two-way repeated-measures ANOVA followed by a Sidak’s multiple comparison test, while an unpaired *t*-test was used for all other experiments to determine significance. Asterisks denote significance (**P < 0.01). n, number of individual mosquitoes examined. Data analysis was performed using GraphPad Prism 7.

Previous studies have argued that bacteria present in the midgut microbiota may contribute to immune responses initiated in the mosquito following *Plasmodium* infection (Dong et al., 2009; Rodrigues et al., 2010; Barletta et al., 2019), supporting that bacteria may contribute to the increase in *UO* expression following *Plasmodium* infection. This is further supported by data showing that bacterial infections upregulate *UO* expression in *Drosophila* (Chambers et al., 2012). Therefore, we wanted to explore whether the microbiota contributed to the differences in uric acid metabolism following infection (Figure 1). To approach this question, we first examined 16s rRNA expression, which serves as a proxy to assess bacteria numbers (Blumberg et al., 2013; Smith et al., 2015; Reynolds et al., 2020a), in naïve blood-fed and *Plasmodium*-infected blood-fed mosquitoes. Both blood-fed and infected mosquitoes displayed increased bacterial proliferation when compared to sugar-fed (NBF) mosquitoes, although infection status had no effect on bacteria numbers (Figure 4A). We next examined whether aseptic mosquitoes treated with antibiotics displayed any differences in uric acid metabolism. While bacteria numbers were significantly reduced following antibiotic treatment (Figure 4B), aseptic mosquitoes displayed no difference in *UO* expression (Figure 4C), or uric acid production (Figure 4D). Therefore, our results suggest that the midgut microbiota have no influence on uric acid metabolism, arguing that *Plasmodium* infection is solely responsible for the changes to mosquito physiology that result in the reduction of uric acid.

## Discussion

Following blood-feeding, the conversion of blood-derived amino acids into lipids and carbohydrates is an essential physiological process needed for egg production. During this process, amino acids are deaminated, resulting in the production of toxic nitrogenous waste that must be excreted by the mosquito vector as uric acid, urea, or as free ammonia (Scaraffia, 2016). While uric acid can be excreted without further modification, uric acid can also be metabolized into allantoin, allantoic acid, and ultimately to urea through several enzymes involved in a uricolytic pathway (Scaraffia et al., 2008; Isoe and Scaraffia, 2013).

**FIGURE 5:**
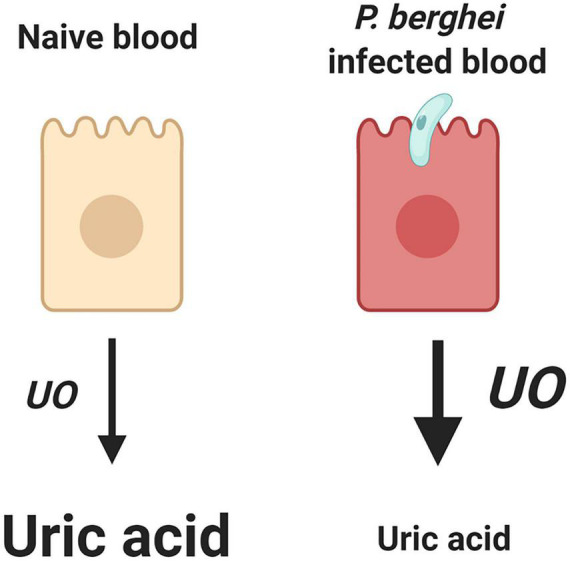
Summary of uric acid metabolism in response to a naïve or *P. berghei-*infected blood meal. We demonstrate that the mosquito host modulates levels of uric acid through the regulation of urate oxidase (*UO*), where upon infection *UO* expression is increased, resulting in less uric acid, which consequently limits malaria parasite survival. Created with BioRender.com.

Here, we demonstrate that *An. gambiae* modulate uric acid levels through the regulation of *UO* expression in response to *P. berghei* infection. Although the mechanism is currently unknown, our data suggest that the mosquito host responds to parasite infection by upregulating *UO* expression, the initial enzyme responsible for uric acid degradation (Scaraffia et al., 2008; Isoe and Scaraffia, 2013). As a result, uric acid levels are decreased, creating an unfavorable environment for *Plasmodium* survival in the mosquito host (summarized in Figure 5). Similar physiological mechanisms have also been described in *Drosophila*, where uric acid levels were decreased following the induction of *UO* expression in response to bacterial challenge (Lang et al., 2019), suggesting that the manipulation of uric acid metabolism is an important defense mechanism of insects against pathogen infection.

Uric acid has previously been described as a potent antioxidant across species, scavenging free radicals to maintain redox homeostasis (Ames et al., 1981; Hilliker et al., 1992). With the production of reactive oxygen and nitrogen metabolites in mosquitoes following blood meal digestion (Sadrzadeh et al., 1984; DeJong et al., 2007; Bottino-Rojas et al., 2015) or in response to *Plasmodium* infection (Luckhart et al., 1998; Kumar et al., 2003; Molina-Cruz et al., 2008), it is not surprising that mosquitoes are able to modify levels of uric acid and other detoxifying enzymes (such as superoxide dismutase, peroxidases, and catalase) to maintain physiological homeostasis. Previous studies have demonstrated that the supplementation of uric acid can improve *Plasmodium* oocyst survival (Peterson et al., 2007; Molina-Cruz et al., 2008), similar to our results in which *UO*, the enzyme responsible for the degradation of uric acid, was silenced *via* RNAi.

Previous studies have suggested that increased oxidative stress can negatively influence mosquito fecundity (DeJong et al., 2007; Champion and Xu, 2018). Although we did not examine the influence of *UO*-silencing on mosquito fecundity in this study, the reduction in uric acid levels following malaria parasite infection may contribute to the lower fecundity of mosquitoes infected with *P. berghei* (Hogg and Hurd, 1995, 1997; Ahmed et al., 2002). This is supported by a recent study in which reduced uric acid levels impaired egg production in kissing bugs (Gandara et al., 2021). As a result, further experiments are required to more closely examine the influence of uric acid levels on mosquito fecundity. In addition, potential differences between *P. berghei* and *P. falciparum* infection may have different impacts on mosquito physiology, thus warranting further exploration.

While previous studies in *Drosophila* (Chambers et al., 2012) and *Anopheles coluzzii* (Chabanol et al., 2020) have, respectively suggested uric acid production and nitrogen metabolism are influenced by bacteria, our experiments using the administration of antibiotics to deplete the microbiota did not alter *UO* expression or uric acid metabolism. These results suggest that the changes in uric acid production following *Plasmodium* infection are independent of the microbiota and are produced by yet unknown mechanisms in response to malaria parasite challenge.

Additional components of the uricolytic pathway may also influence mosquito physiology and pathogen infection, yet have not been fully explored. Previous studies in *An. stephensi* demonstrate that allantoin, a downstream product of uric acid metabolism, does not influence malaria parasite infection (Peterson et al., 2007). However, the role of allantoic acid on parasite survival has not previously been examined. Our data demonstrating the increased expression of *ALLC* following *P. berghei* challenge suggests that allantoic acid may also influence parasite infection and is a target of future work.

In summary, we demonstrate that the mosquito host modulates uric acid metabolism in response to malaria parasite infection through the regulation of urate oxidase (UO) activity, limiting *P. berghei* survival. These new insights connect previous observations of the role of uric acid as a determinant of malaria parasite infection, highlighting the importance of uric acid metabolism in defining mosquito vector competence to *Plasmodium* infection.

## Data Availability Statement

The original contributions presented in the study are included in the article/Supplementary Material, further inquiries can be directed to the corresponding authors.

## Ethics Statement

The animal study was reviewed and approved by the Animal Care and Use Committee at Iowa State University.

## Author Contributions

HK performed the experiments. HK and RS conceived the experiments, analyzed the data, and wrote the manuscript. Both authors contributed to the article and approved the submitted version.

## Conflict of Interest

The authors declare that the research was conducted in the absence of any commercial or financial relationships that could be construed as a potential conflict of interest.

## Publisher’s Note

All claims expressed in this article are solely those of the authors and do not necessarily represent those of their affiliated organizations, or those of the publisher, the editors and the reviewers. Any product that may be evaluated in this article, or claim that may be made by its manufacturer, is not guaranteed or endorsed by the publisher.

## Footnotes

1
http://www.dkfz.de/signaling/e-rnai3/idseq.php
