# Supplementary material for: Anopheles gambiae Actively Metabolizes Uric Acid Following Plasmodium Infection to Limit Malaria Parasite Survival
Source: Front Physiol. 2022 Jan 24;12:821869. doi: 10.3389/fphys.2021.821869 (PMC8818946; doi:10.3389/fphys.2021.821869)
Supplement: Supplementary file 1 [file Table_1.DOCX]

| **Table S1.**  **Primers used for dsRNA synthesis and qRT-PCR** | | | | |
| --- | --- | --- | --- | --- |
| **Primer name** | | **Sequences (5’-3’)** | **Gene ID** | |
| **For dsRNA synthesis** | | | | |
| Anoga-UOT7F  Anoga-UOT7R | TAATACGACTCACTATAGGGGACTAGCAGCCGTCCAGTCA  TAATACGACTCACTATAGGGCTGATCAGGAAGGGGGAGTT | | | AGAP008440 |
| GFP-T7F  GFP-T7R | TTAATACGACTCACTATAGGGAGAATGGTGAGCAAGGGCGAGGAGCTGT  TTAATACGACTCACTATAGGGAGATTACTTGTACAGCTCGTCCATGCC | | |  |
| **For qRT-PCR** |  | | |  |
| Anoga-XDHqF | AATTCACTGCTCCGAAACGG | | | AGAP013758 |
| Anoga-XDHqR | GTAGGGCTCTAGGCGTGATT | | |  |
| Anoga-UOqF  Anoga-UOqR | GTCCAGAACACGCTCTACCT  TTCACCACCTTCGGGAACTT | | | AGAP008440 |
| Anoga-ALLNqF | TCACTGATCTATCAACGCACG | | | AGAP000239 |
| Anoga-ALLNqR | TCTTCAACCGATATGCTTCCTG | | |  |
| Anoga-ALLCqF | TTGGATGCTGCTGATGGAGA | | | AGAP004501 |
| Anoga-ALLCqF | TAATACGCACCAGTGAGGCT | | |  |
| Anoga-rps7qF  Anoga-rps7qR | ACCACCATCGAACACAAAGTTGACACT  CTCCGATCTTTCACATTCCAGTAGCAC | | | AGAP010592 |
| Universal bacterial 16s F | TCCTACGGGAGGCAGCAGT | | |  |
| Universal bacterial 16s R | GGACTACCAGGGTATCTAATCCTGTT | | |  |
